# Isolation of a Pluripotent Neural Stem Cell from the Embryonic Bovine Brain

**DOI:** 10.3390/ijms16035990

**Published:** 2015-03-13

**Authors:** Yuhua Gao, Xiangchen Li, Dong Zheng, Weijun Guan, Yuehui Ma

**Affiliations:** 1 Institute of Animal Sciences, Chinese Academy of Agricultural Sciences, Beijing 100193, China; E-Mails: gyhann@hotmail.com (Y.G.); nobelli@126.com (X.L.); 2 College of Wildlife Resources, Northeast Forestry University, Harbin 150040, China; E-Mail: profzhengdong@yeah.net

**Keywords:** bovine, neural stem cell, differentiation

## Abstract

We recently isolated stem cells derived from the brain of a bovine fetus, utilizing a particular mechanical separation method. After improving our experimental conditions, we obtained neural stem cells using an optimized culture medium system. The cells were expanded, established in continuous cell culture and used for immunofluorescence cytochemistry. RT-PCR showed that embryonic neural stem cells (NSCs) not only expresses the protein Sox2, Nestin but also Pax6, Musashi proteins and were differentiated into the three classical neuronal phenotypes (neurons, astrocytes, and oligodendrocytes).

## 1. Introduction

The first demonstration of neurogenesis in non-mammal vertebrates came from animals such as birds or lizards. Neurogenesis was also confirmed to occur in adult mammals, like mice and rats, and, finally, in primates and humans.

Most cells in the nervous system are born during the embryonic and early postpartum periods, but new neurons are continuously added in certain regions of the adult mammalian brain [1]. These neurons are thought to derive from a population of stem cells, and it was shown by Weiss [2] that neural stem cells taken from the brain can be propagated *in* *vitro*. These cells have the capacity for self-renewal and can generate into the major classes of central nervous system cell types, that is, neurons, astrocytes, and oligodendrocytes [3,4]. Neural stem cells (NSCs) can be isolated from the walls of the ventricular system of the central nervous system as well as from the hippocampus [5,6,7,8].

Current research of stem cells focuses on humans, mice and rabbits but little research has been performed on bovine. In this study, we carried out a pilot study on the separation, culture, and differentiation potential of bovine NSCs.

## 2. Results and Discussion

### 2.1. Isolation, Culture, and Morphology of NSCs

**Figure 1:**
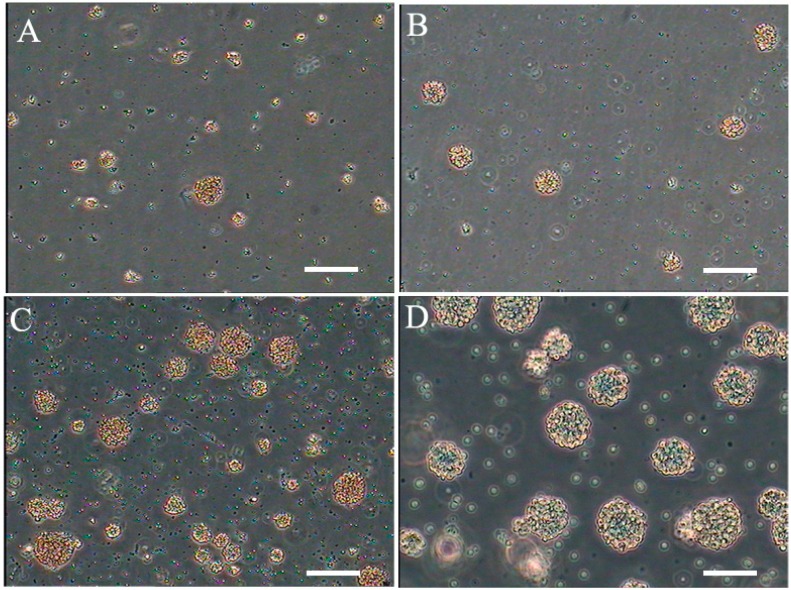
Floating neural spheres generated from bovine brain in different medium. (**A**) medium I; (**B**) medium II; (**C**) NSCs cultured in medium III 7 days and (**D**) cultured in medium III 20 days. Scale bar = 200 μm.

Cells derived from the dissociation of embryonic forebrain samples (described in the Experimental Section) were suspended in medium I (Figure 1A), medium II (Figure 1B) and medium III (Figure 1C), respectively. Assembly of neurospheres was very slow and the spheres were quite small. In all cultures, many single cells attached to the flasks; however, as the cells proliferated into small clusters, they detached from the plastic and floated in suspension. Figure 1C illustrates representative clusters from bovine fetus brain at seven days. The clusters continued to increase significantly over a period of 20 days (Figure 1D), depending on growth conditions. After clusters formed, the cells were passaged by mechanical dissociation into a single cell suspension. The cells were reseeded and passaged continuously for up to 10–15 passages, and the cells remained viable.

### 2.2. Optimization of NSCs

There was no significant difference between culture systems I and II (*p* > 0.05). The generation time was about two weeks for both systems. Culture system III and the other culture systems were significantly different and resulted in a generation time of about seven days (*p* < 0.05) (Figure 1B). These results indicated that epidermal growth factor (EGF) and fibroblast growth factor-2 (FGF-2) promote NSC proliferation, and culture system III is suitable for expansion of NSCs.

No obvious morphological differences were observed among passages, and the characteristics of the cells were stable after passaging. The cells were cultured to passage 10 and showed the representative appearance of senescence, such as dispersing and karyopyknosis in most cells.

### 2.3. Characterization of NSCs

#### 2.3.1. Markers of NSCs

**Figure 2:**
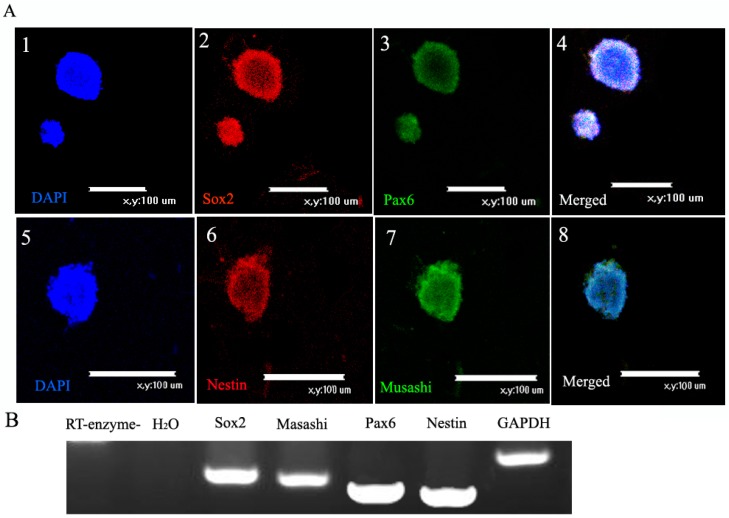
Markers of neural stem cells (NSCs). (**A**) Immunofluorescence of NSCs: (**1**) Nuclear counterstaining (blue) with DAPI; (**2**) immunofluorescence staining of the marker Sox2 (red) and (**3**) Pax6 (green); (**4**) Merged picture to show Sox2 and Pax6 co-expressed in NSCs. Scale bar = 100 μm (**1**–**4**); (**5**) Nuclear counterstaining (blue) with DAPI, (**6**) immunofluorescence staining of the marker Nestin (red) and (**7**) Musashi (green); (**8**) Merged picture to show Nestin and Musashi co-expressed in NSCs. Scale bar = 100 μm (**5**–**8**); and (**B**) DNA electrophoretic analysis of cell marker. RT-PCR analysis of NSCs indicated expression of Sox2, Musashi, Pax6 and Nestin. GAPDH served as the internal control.

We detected markers of NSCs by immunofluorescence and RT-PCR. The immunofluorescence (Figure 2A) and RT-PCR (Figure 2B) results showed that NSCs expressed Sox2, Nestin, Pax6, and Musashi. There were no apparent differences in these markers at different passages.

#### 2.3.2. Neurogenic Differentiation of NSCs

**Figure 3:**
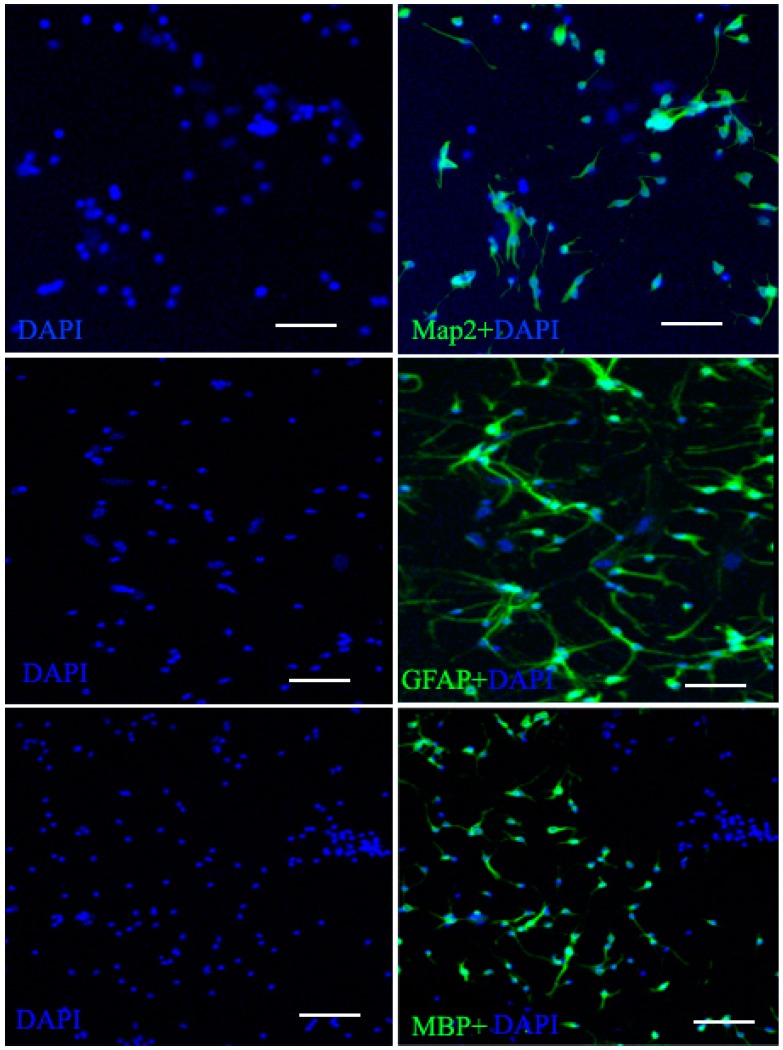
Neurogenic cell differentiation of the NSCs. After two weeks induction, NSCs neurogenic differentiation occurred gradually and neurogenic processes progressively elongated. The neurogenic cells markers MBP, (oligodendrocyte cell marker) Map2 (neuronal cell marker) and GFAP (astrocyte cell marker) were present in the induction group by immunofluorescence. Scale bar = 100 μm.

Under differentiation and poly-l-lysine-coated conditions, the cells immediately adhered to the substrate and began to differentiate, and after several days *in vitro*, different morphologies were apparent, including large flat cells and small bipolar and multipolar cells. Within 7 to 10 days the cells had proliferated to create a monolayer. Three cells were evaluated for expression of Map2 (neuron cells marker), GFAP (astrocyte cell marker), and MBP (oligodendrocyte cell marker) (Figure 3).

**Figure 4:**
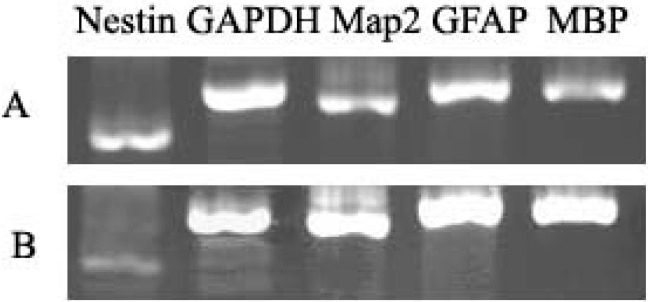
DNA electrophoretic analysis of neurogenic cell marker. RT-PCR analysis of induction group indicated higher expression levels of MBP, Map2 and GFAP. (**A**) Undifferentiated NSCs; and (**B**) Differentiated cells.

RT-PCR was used to identify induced cells, the GFAP, Map-2, MBP and Nestin were all positive in undifferentiated NSCs (Figure 4), the level of GFAP, Map2, and MBP were higher than undifferentiated NSCs but Nestin was lower than undifferentiated NSCs. Results suggest that the NSCs have the potential to differentiate into three types of neurogenic cells.

### 2.4. Discussion

The bovine (*Bos taurus*) possesses a large and highly gyrencephalic brain and the long gestation period (41 weeks) is comparable to human pregnancy (38–40 weeks). Bovine neural cells and specific *in vitro* cell cultures could be an alternative in comparative neuroscience and in neurodegenerative research, useful for studying development of normal and altered circuitry in a long gestation mammalian species. Use of bovine tissues would promote a substantial reduction in the use of laboratory animals [9,10,11,12].

Initially, cells were seeded at 1000 viable cells/cm^2^, final enrichment steps at one cell per flask. The ratio of number of spheres formed after 7 days *in vitro* to number of cells plated is the NSC frequency. Subsequent passaging of primary spheres was performed by mechanically dissociating collected spheres (centrifuged for 10 min at 100× *g*) into a single cell suspension and replacing in medium containing EGF, FGF-2 and N2 as Experiment section.

EGF and FGF-2 are expressed in the neurogenic areas of the adult CNS [13], suggesting that these trophic factors may also regulate adult neurogenesis *in vivo*. In support of this contention, EGF administered by chronic infusions into the lateral ventricle of adult rats stimulates the proliferation of neural stem cells in the subventricular zone (SVZ). EGF belongs to the EGF family of trophic factors, including transforming growth factor-α (TGF-α). The EGF family of trophic factor signals through the EGF receptor, a tyrosine kinase receptor encoded by the *ErbB* gene [14]. A reduction of the dividing progenitor cell population of the SVZ and the olfactory bulb (OB) were reported in FGF-2 knockout mice compared to littermate control [15]. This further supported the importance of FGF-2 in the regulation of neurogenesis *in vivo*. To assess whether EGF and FGF-2 were necessary for proliferation of the NSCs, trophic factors were removed [16]. We observed the slow proliferation of NSCs cultured in the system I of our study; the generation time was longer than for system III, the cells were very robust and gathered into the neural sphere. N2 supplement contains insulin, transferrin, sodium selenite, putrescine and progesterone in PBS [17]. It was critically important for neural cells, and neural stem cells in N2-free medium to develop into spheres very slowly, and the neurosphere was smaller than that in culture system III, which contained the N2 supplement. Thus we chose the culture system III; These multipotent cells are maintained as nonadherent clusters, in the presence of the mitogen EGF and FGF. These clusters can be expanded in culture for extended periods of time (passage10).

We isolated and expanded the bovine stem cells in the presence of EGF and FGF-2 [18,19,20].

In these culture systems, removal of mitogens and the addition of serum will result in the differentiation of the progenitor cells into neurons and glia. In such conditions, neural stem cells have the tendency to differentiate into neurons and glial cells. In this experiment, D/F12 with 5% FBS, neural stem cells can differentiate into two important types of cells, star-shaped glial cells and neurons. This is a preliminary study on the function and nature of differentiated cells, because the final application of neural stems cells is differentiation and function of the differentiated state. Thus, studies on neural stem cells under various conditions, and with various factors, alone or in combination, and the means to obtain a higher level of target cell cloning and expression of genes and proteins in this process will be the focus of future investigations.

## 3. Experimental Section

### 3.1. Reagents and Animals

All cell culture medium and supplements were purchased from Sigma-Aldrich (St. Louis, MO, USA), unless stated otherwise.

Bovine placenta samples collected under sterile conditions after caesarean section (at 14–18 weeks gestation) were provided by the Chinese Academy of Agricultural Sciences farm. Animal experiments were performed in accordance with the guidelines established by the Institutional Animal Care and Use Committee at the Chinese Academy of Agricultural Sciences.

### 3.2. Isolation of NSCs from Bovine Brain Embryo

According to previous research [21,22], the NSCs culture medium was not suitable for bovine; thus we optimized the best culture medium conditions for NSCs.

The brain was dissociated from bovine fetus. The brain, almost soft, which contains vessels and blood, was washed several times to remove blood in 1 M cold PBS. The tissue was transferred to Dulbecco’s modified Eagle’s medium/Ham’s F-12 (D/F12) medium containing plate and was cultivated into a monoplast suspension after repetitive beat by pipette and mechanical separation, and then single cells were plated at 1000 cells/cm^2^ in untreated 25-cm^2^ tissue culture flasks, cultured in completed neural medium as described below.

### 3.3. Optimization of Cell Culture Systems for NSCs

NSCs culture at passage 2 was assessed in three culture systems: culture system I (D/F12 + 1% (*v*/*v*) N2 supplement (Gibco, Carslbad, CA, USA) + 2 μg/mL heparin), culture system II (D/F12 + 20 ng/mL EGF (Invitrogen, San Diego, CA, USA), 20 ng/mL FGF-2 (Invitrogen) + 2 μg/mL heparin), and culture system III (D/F12 + 1% N2 supplement + 20 ng/mL EGF+ 20 ng/mL FGF-2 + 2 μg/mL heparin). The cells were cultured further and the generation time in each culture system was counted three times. Culture system III was named neural medium and subsequently used to culture NSCs.

After 7–10 days, cultures were harvested, mechanically dissociated, and replated under the same conditions. After performing this procedure twice to eliminate short-term dividing precursors, bulk cultures were generated by passaging cells at higher density (10^4^ cells/cm^2^) every 7–10 days in the same growth medium.

### 3.4. Marker of NSCs

NSCs were seeded in 100 μg/mL poly-l-Lysine coated coverslips and incubated for 24 h, then incubated with 4% paraformaldehyde for 15 min and then washed three times in PBS (5 min each). Cells were permeabilized with 0.2% Triton X-100 for 20 min and then washed three times (5 min each) in PBS. The cells were blocked with 10% normal goat serum (Santa Cruz Biotechnology, Santa Cruz, CA, USA) for 30 min and then incubated at room temperature for 1 h in PBS containing the following antibodies: mouse anti-Nestin (1:100; Abcam, Cambridge, MA, USA), mouse anti-Pax6 (1:200; Abcam), Rabbit anti-Sox2 (1:200; Abcam), rabbit anti-Musashi (1:100; Bioss, Beijing, China), Chicken anti-GFAP (IgY; 1:200; Bioss), rabbit anti-MBP (1:200; Bioss), rabbit anti-Map2 (1:200; Santa Cruz Biotechnology). Then, the cells were washed three times with PBS and then incubated in PBS containing secondary antibodies at 37 °C for 1 h. Secondary antibodies were Cy3-conjugated goat anti-mouse IgG, goat anti rabbit IgG, FITC-conjugated rat anti-chicken IgY, and donkey anti-rabbit IgG (Santa Cruz Biotechnology).

Cells were examined under a TE-2000-E inverted fluorescence microscope (Nikon, Yokohama, Japan). Cells were counterstained with DAPI (Sigma-Aldrich).

### 3.5. RT-PCR Assays

RNA was isolated from cells using Trizol reagent (Invitrogen). cDNA was synthesized using a reverse transcription system (Takara, Dalian, China) and amplified by PCR using specific primers (Table 1). PCR products were visualized by 3% agarose gel electrophoresis.

**Table 1:** Primer sequences used in PCR assay.

Gene	Primer Sequence	*T*_m_ (°C)	Fragment Size (bp)
*Sox 2*	F: 5'-TCCTATTCTCAGCAGGGCAC-3'	61	217
	R: 5'-AGTGCTGGGACATGTGAAGT-3'		
*GAPDH*	F: 5'-GGCAAGTTCAACGGCACAGTCA-3'	58	364
	R: 5'-TAAGTCCCTCCACGATGCCAAAG-3'		
*GFAP*	F: 5'-GTCGTGGGTGAGCAGTTACA-3'	58	341
	R: 5'-CTAAAACACGGGGAGGTGGG-3'		
*MBP*	F: 5'-CAGAGACACTGGCATCCTCG-3'	60	350
	R: 5'-CAGACGCTCTGCCTCCATAG-3'		
*MAP2*	F: 5'-CGCCAATGGATTCCCCTACA-3'	54	322
	R: 5'-TTCCTCCACTGGGACAGTCT-3'		
*Musashi*	F: 5'-GTCTCGAGTCATGCCCTACG-3'	55	223
	F: 5'-CATGGGTCCATAAGCGGTGA-3'		

*T*_m_: melting temperature; F: forward; R: reverse.

### 3.6. Differentiation

To exclude possible cellular aggregates during explant culture, the secondary spheres were chosen for cloning and differentiation analysis. Five-hundred single secondary spheres were placed on poly-l-lysine in six-well plate chambers and were covered with D/F12 containing 5% fetal bovine serum (FBS; Gibco), 20 ng/mL EGF, 20 ng/mL bFGF and 1% N2 supplement. Supplement and l-glutamine were added for 3 h in an incubator to allow the spheres to attach on coverslips. Phenotype differentiation was induced by a combination of neurotrophins, including 10 ng/mL brain-derived neurotrophic factor (GDNF) (Invitrogen), 10 ng/mL neurotrophin (NT)-3 and cultured for 12 days, with the medium changed every three days.

## 4. Conclusions

In conclusion, this study establishes an optimized method for the isolation and culture of bovine NSCs as suggested by characterization of cell morphology, cell markers and biological features. We also demonstrate that NSCs can be induced to differentiate into three types of neurogenic cells, which supports the notion that the NSCs retained multipotency. This study not only provides a technological platform for the establishment of a bovine NSCs bank, but also proposes a new method to show an animal model applied to human.

## References

[1] Altman J., Das G.D. (1965). Autoradiographic and histological evidence of postnatal hippocampal neurogenesis in rats. J. Comp. Neurol..

[2] Weiss S., Dunne C., Hewson J., Wohl C., Wheatley M., Peterson A.C., Reynolds B.A. (1996). Multipotent CNS stem cells are present in the adult mammalian spinal cord and ventricular neuroaxis. J. Neurosci..

[3] Imura T., Kornblum H.I., Sofroniew M.V. (2003). The predominant neural stem cell isolated from postnatal and adult forebrain but not early embryonic forebrain expresses GFAP. J. Neurosci..

[4] Wen C.M., Cheng Y.H., Huang Y.F., Wang C.S. (2008). Isolation and characterization of a neural progenitor cell line from tilapia brain. Comp. Biochem. Physiol. A Mol. Integr. Physiol..

[5] Morrison S.J., Shah N.M., Anderson D.J. (1997). Regulatory mechanisms in stem cell biology. Cell.

[6] Lois C., Alvarez-Buylla A. (1993). Proliferating subventricular zone cells in the adult mammalian forebrain can differentiate into neurons and glia. Proc. Natl. Acad. Sci. USA.

[7] Palmer T.D., Takahashi J., Gage F.H. (1997). The adult rat hippocampus contains primordial neural stem cells. Mol. Cell Neurosci..

[8] Van Strien M.E., Sluijs J.A., Reynolds B.A., Steindler D.A., Aronica E., Hol E.M. (2014). Isolation of neural progenitor cells from the human adult subventricular zone based on expression of the cell surface marker CD271. Stem Cells Transl. Med..

[9] Liu Y., Chen F., Liu W., Cui L., Shang Q., Xia W., Wang J., Cui Y., Yang G., Liu D. (2002). Repairing large porcine full-thickness defects of articular cartilage using autologous chondrocyte-engineered cartilage. Tissue Eng..

[10] Bosnakovski D., Mizuno M., Kim G., Ishiguro T., Okumura M., Iwanaga T., Kadosawa T., Fujinaga T. (2004). Chondrogenic differentiation of bovine bone marrow mesenchymal stem cells in pellet cultural system. Exp. Hematol..

[11] Kon E., Muraglia A., Corsi A., Bianco P., Marcacci M., Martin I., Boyde A., Ruspantini I., Chistolini P., Rocca M. (2000). Autologous bone marrow stromal cells loaded onto porous hydroxyapatite ceramic accelerate bone repair in critical-size defects of sheep long bones. J. Biomed. Mater. Res..

[12] Peruffo A., Cozzi B. (2014). Bovine brain: An *in vitro* translational model in developmental neuroscience and neurodegenerative research. Front. Pediatr..

[13] Emoto N., Gonzalez A.M., Walicke P.A., Wada E., Simmons D.M., Shimasaki S., Baird A. (1989). Basic fibroblast growth factor (FGF) in the central nervous system: Identification of specific loci of basic FGF expression in the rat brain. Growth Factors.

[14] Riese D.J., Stern D.F. (1998). Specificity within the EGF family/ErbB receptor family signaling network. Bioessays.

[15] Zheng W., Nowakowski R.S., Vaccarino F.M. (2004). Fibroblast growth factor 2 is required for maintaining the neural stem cell pool in the mouse brain subventricular zone. Dev. Neurosci..

[16] Vescovi A.L., Parati E.A., Gritti A., Poulin P., Ferrario M., Wanke E., Frolichsthal-Schoeller P., Cova L., Arcellana-Panlilio M., Colombo A. (1999). Isolation and cloning of multipotential stem cells from the embryonic human CNS and establishment of transplantable human neural stem cell lines by epigenetic stimulation. Exp. Neurol..

[17] Park E.M., Joh T.H., Volpe B.T., Chu C.K., Song G., Cho S. (2004). A neuroprotective role of extracellular signal-regulated kinase in *N*-acetyl-*O*-methyldopamine-treated hippocampal neurons after exposure to *in vitro* and *in vivo* ischemia. Neuroscience.

[18] Tropepe V., Sibilia M., Ciruna B.G., Rossant J., Wagner E.F., van der Kooy D. (1999). Distinct neural stem cells proliferate in response to EGF and FGF in the developing mouse telencephalon. Dev. Biol..

[19] Sutterlin P., Williams E.J., Chambers D., Saraf K., von Schack D., Reisenberg M., Doherty P., Williams G. (2013). The molecular basis of the cooperation between EGF, FGF and eCB receptors in the regulation of neural stem cell function. Mol. Cell. Neurosci..

[20] Supeno N.E., Pati S., Hadi R.A., Ghani A.R., Mustafa Z., Abdullah J.M., Idris F.M., Han X., Jaafar H. (2013). IGF-1 acts as controlling switch for long-term proliferation and maintenance of EGF/FGF-responsive striatal neural stem cells. Int. J. Med. Sci..

[21] Pagano S.F., Impagnatiello F., Girelli M., Cova L., Grioni E., Onofri M., Cavallaro M., Etteri S., Vitello F., Giombini S. (2000). Isolation and characterization of neural stem cells from the adult human olfactory bulb. Stem Cells.

[22] Walker T.L., Kempermann G. (2014). One mouse, two cultures: Isolation and culture of adult neural stem cells from the two neurogenic zones of individual mice. J. Vis. Exp..

